# Individual and contextual factors of influence on adherence to antiretrovirals among people attending public clinics in Rio de Janeiro, Brazil

**DOI:** 10.1186/1471-2458-13-574

**Published:** 2013-06-13

**Authors:** Homaira Hanif, Francisco I Bastos, Monica Malta, Neilane Bertoni, Pamela J Surkan, Peter J Winch, Deanna Kerrigan

**Affiliations:** 1 Johns Hopkins Bloomberg School of Public Health, Department of International Health, Baltimore, USA; 2 Oswaldo Cruz Foundation, Health Information, Rio de Janeiro, Brazil; 3 Oswaldo Cruz Foundation, FIOCRUZ, DCS/ENSP, Rio de Janeiro, Brazil; 4 Johns Hopkins Bloomberg School of Public Health, Department of Health, Behavior and Society, Baltimore, USA

**Keywords:** HIV, Adherence, Antiretrovirals, Mental health, Patient care

## Abstract

**Background:**

There are inconsistencies in the determinants of adherence to antiretrovirals (ARVs) across settings as well as a lack of studies that take into consideration factors beyond the individual level. This makes it necessary to examine factors holistically in multiple settings and populations while taking into consideration the particularities of each context, in order to understand the patterns of ARV adherence. This research explored ARV adherence and individual, relational and environmental-structural factors.

**Methods:**

A cross-sectional survey was conducted from August 2008 through July 2009 among participants currently on ARVs recruited from 6 public health clinics, selected to maximize diversity in terms of caseload and location, representing the range of clinics within Rio de Janeiro city, Brazil. Multivariate logistic regression analysis was used to assess the association between our multilevel factors with ARV adherence among participants with complete cases (n = 632).

**Results:**

Eighty-four percent of respondents reported adherence to all of their ARV doses in the last 4 days. Of the socio-demographic variables, those who had one child were positively associated with adherence (AOR 2.29 CI [1.33-3.94]). On the relational level, those with high social support (AOR 2.85 CI [1.50-5.41]) were positively associated with adherence to ARVs. On the environmental-structural level, we found gender was significant with women negatively associated with adherence to ARVs (AOR 0.58 CI [0.38-0.88]) while those with a high asset index (AOR 2.47 CI [1.79-3.40]) were positively associated with adherence to ARVs.

**Conclusions:**

This research highlights the importance of examining the multiple levels of influence on ARV adherence. Intervention research in lower and middle-income settings should address and evaluate the impact of attending to both gender and economic inequalities to improve ARV adherence, as well as relational areas such as the provision of social support.

## Background

Brazil is a pioneer in providing universal free access to antiretrovirals (ARVs). Despite continued concerns about the financial sustainability of the ARV program, Brazil has been able to implement a successful program with mortality and morbidity among people living with HIV (PLWH) dropping drastically since the mid-1990s [1,2]. While there have been concerns that the roll-out of ARVs in resource-limited settings would be associated with lower adherence to ARVs, leading to drug resistance, data show that adherence levels in resource-poor settings are similar to those in resource-rich settings [3,4]. Brazil has demonstrated that ARV therapy can be delivered in a resource limited setting without creating widespread transmissions of resistant strains [5-8]. The sustainability of Brazil’s universal provision of ARVs is in question as case management becomes more complex with more time on ARVs resulting in more side effects, an increase in ARV costs due to scaling up treatment, and the need to move from first to second and third line treatment [9]. Since a certain amount of drug resistance can be attributed to low medication adherence, improving adherence is an important factor in the overall sustainability of the program. In addition, with the global roll-out of ARVs ongoing, it is important to examine issues surrounding medication adherence in lower and middle-income countries.

The introduction of combination ARVs changed the progression of HIV to AIDS by increasing life expectancy and making HIV a chronic disease requiring daily medications [10-12]. While almost perfect adherence to the complex regimen is required to maintain viral suppression, a recent meta-analysis found an average rate of adherence to ARVs at 62 percent worldwide [13]. Adherence to ARVs has become an important area of research because it is a strong predictor for progression to AIDS; furthermore, adherence may be improved through carefully planned interventions [3,14-16]. While significant research has been conducted on factors associated with adherence to ARVs, a limited number of factors have been consistently identified across multiple settings [17].

Taking into account the complex interplay of factors determining adherence, researchers have categorized variables into groups such as: patient factors, medication regimen, disease characteristics, patient-provider relationship and the clinical setting [18,19]. Sociodemographic variables have not been shown to consistently predict ARV or other medication adherence; [20,21] though being male, white, older, having higher income and higher literacy have more often been associated with better adherence [22-24]. Psychosocial factors such as depression and psychiatric disorders have been associated with poor medication adherence [25-29]. Depression is associated with worse outcomes, and has been associated with an accelerated decline in CD4 count and HIV disease progression [30]. With regard to relational factors, social support has been found to be a significant determinant of adherence in several studies, with low levels of social support consistently predicting nonadherence [25,31-34]. A systematic review of available evidence in the PubMed database on socioeconomic status (SES) and adherence to ARVs did not find a consistent association but noted a positive trend between higher levels of SES and adherence [35].

The literature on adherence in Brazil is not extensive and is limited by varying definitions of adherence and populations sampled. While this is the case in the general adherence literature, these limitations make it difficult to observe patterns of adherence and limit generalizability for Brazil as a whole. Studies in Brazil have identified a range of adherence patterns, from 52 to 83 percent adherence, potentially due to how adherence was measured [7,36]. The variables found to be important are similar to those previously discussed including a combination of patient, medication and clinic level factors. Some of these factors are adherence self-efficacy, frequency of dosing, medication beliefs, forgetfulness, alcohol use, depression, unemployment and misunderstandings about the prescription [6,37-39]. One of the largest studies was conducted by Nemes et al. [40], which included patients under follow-up in health units from 7 Brazilian states with a sample size of 1,972. They found adherence prevalence was 75 percent with non-adherence predicted by low levels of formal education, complex regimens, and number of missed appointments. Another study in Rio de Janeiro had high self-reported adherence at 82 percent, with self-efficacy, sexual orientation, loss of appetite and the doctor-patient relationship found to be significantly associated with adherence [8]. Research among low-income women on ARVs in São Paulo found women had unmet care needs regarding medical information and psychological support, indicating the necessity of better integrating services and increasing focus on women’s needs [41].

There have been a number of studies examining adherence to ARVs across different countries and populations and several systematic reviews regarding the determinants of adherence to ARVs [17,32]. The many inconsistencies in the determinants of adherence to ARVs have been attributed to the differences among populations, statistical power and sampling methodology, the predictors included and how they were conceptualized and measured, and how adherence itself was measured. This makes it necessary to examine factors holistically in multiple settings and populations while taking into consideration the particularities of each context, in order to understand the patterns of ARV adherence.

The research on adherence to ARVs in Brazil has focused on patient factors and medication regimen with some research on the clinic setting. In addition, most research has been conducted in referral centers, such as University hospitals and research centers. The majority of PLWH in Brazil access care through the public clinics, which are often located in impoverished communities and serve the poor to low-middle class populations. This research contributes to the understanding of ARV adherence among men and women from a heterogeneous sample of public clinics. We contextualized adherence behavior by examining multiple levels of influence such as environmental-structural (economic and gender), relational (social support), and individual (sociodemographic and psychosocial) factors. The aim is to understand the factors that facilitate or impede adherence to develop more comprehensive care and targeted interventions that better meet the needs of PLWH accessing care through public clinics.

## Methods

### Participants and procedures

This analysis is part of a larger study by the Oswaldo Cruz Foundation focusing on the psychosocial and structural factors associated with the sexual health and wellbeing of people living with HIV/AIDS receiving treatment from public health clinics in Rio de Janeiro city. The study recruited 900 participants, of whom 650 were currently on ARV. This analysis focuses on complete case participants on ARVs (n = 632).

Eligible participants were at least 18 years of age, had a confirmed HIV-positive status, received HIV treatment and care from a public health clinic, and were willing to provide consent. The participants were recruited from six key public health facilities of the total 29 health centers managed by the Rio de Janeiro Health Secretariat. These clinics are located in the urban area of the municipality of Rio. All clinics included in this study are secondary level clinics, which provide care for many different conditions above a primary care clinic. All services are free, including all medications for HIV/AIDS and other chronic conditions such as diabetes and hypertension. Insurance is not required. The six clinics were selected based on caseload (smaller versus larger clinics) and location (geographic and administrative), ensuring a heterogeneous sample representing the range of clinics. These six facilities provide care for approximately 60 percent of the over 14,000 patients living with HIV/AIDS receiving follow-up. The study received ethical approval from the Institutional Review Boards (IRB) of the Johns Hopkins School of Public Health, the Oswaldo Cruz Foundation, the Municipal Health Secretariat, and the National IRB of the Brazilian Ministry of Health.

A pilot test using focus groups was conducted in each clinic before survey implementation to ensure clarity of the questionnaire. Once the questionnaire was finalized, the survey was conducted from August 2008 through July 2009. A trained supervisor recruited participants in the waiting area of the clinics. The purpose of the survey was explained to potential participants and any questions were answered. After oral consent was given, the participant was taken to a private room where a trained interviewer went through the consent form and obtained written consent. The questionnaire took approximately 50 minutes to complete. Interview responses were recorded on Teleform® scannable data forms and reviewed by the interviewer for completeness and consistency prior to the participant’s departure. The forms were scanned for computer entry by the project coordinator.

### Measures

Measures in the survey included sociodemographic variables as well as psychological well-being, measured by anxiety and depression, social support and economic factors. The dependent variable, ARV adherence, and measure details are listed below.

Socio-demographic and Background Variables: Analysis variables included: age, race/ethnicity, education, sexual orientation, relationship status, HIV status of partner, number of children, and length of time on ARVs.

Psychological Well-Being: The Hospital Anxiety and Depression Scale (HAD) is a tested tool to measure psychological well-being over the past week, especially for chronic conditions, and has been used in Brazil [8,39,42]. This scale is composed of 14 questions divided into an anxiety subscale and a depression subscale to identify possible and probable cases of anxiety and depression among non-psychiatric patients. Physical symptoms are not included in this scale (e.g. headaches, insomnia, fatigue). Responses are rated on a 4 point scale (hardly at all to most of the time) and include items such as: “I felt tense or wound-up;” “I enjoyed the things I use to;” “I had worrying thoughts go through my mind;” “I felt cheerful;” and “I could sit at ease and relax.” This study used 11 as the cutoff for possible depression (Cronbach’s alpha = .76) or anxiety (Cronbach’s alpha = .81), respectively [43].

Social support: The social support measure consisted of nine questions from the Medical Outcomes Study (MOS) social support survey developed for chronic conditions [44] which has been validated in Brazil [45], as well as four additional questions to strengthen the tangible support aspect of the measure including perceived support in relation to obtaining medication, monetary aid, housing, and overall satisfaction with support. Respondents answered on a five point scale how often in the last month each kind of support was available to them. The scale had a Cronbach’s alpha of .90 and was categorized into low, medium, and high support based on the distribution.

Gender: Divided into 3 categories: male, female or transgendered persons.

Economic Factors: Personal income (type and amount) and household income was measured as well as having savings. An asset based wealth (SES) index was used based on the IBGE (Brazilian Institute of Geography and Statistics) consisting of ownership of household items such as a radio, TV, refrigerator, etc. The index ranged from 1 to 23 with a median of 7 items and had a Cronbach’s alpha of .77. The index was dichotomized at the median into low and high asset levels.

ARV adherence: A modified AIDS Clinical Trial Group’s (ACTG) questionnaire was used to measure medication adherence [46] which has been used in previous studies in Brazil [47]. The questions focus on recent adherence to minimize recall bias. The outcome adherence was measured by the question: In the last four days, how many days did you take all of your doses of all of your HIV medications? The measure was dichotomized into adherent if the response was 4 days and non-adherent if anything else was chosen.

### Analytic strategy

Data were analyzed using the statistical software Stata11.0 [48]. Descriptive analysis was conducted to examine the distribution of the data such as frequencies, medians, and ranges. Aggregate measures were developed using reliability analysis to assess internal consistency.

Bivariate statistical tests such as t-tests (for continuous variables) and chi-square tests (for categorical variables) were utilized to characterize the direction and significance of the relationship between major study variables, as was bivariate logistic regression. All significant variables of p-value less than 0.10 from the bivariate analysis were included in the multivariate analysis, as well nonsignificant variables hypothesized to be confounders or effect modifiers based on findings from previous research, or included for conceptual purposes. Multivariate logistic regression analysis was conducted to assess the adjusted association between the primary independent variables and the dependent variable, adherence to ARVs. All independent variables were assessed for collinearity by examining their correlation coefficients. Individuals who receive services from the same clinic may be more similar with regard to adherence to ARVs, creating a potential clustering effect. The variance-covariance estimate (VCE) command was used to adjust for this within clinic intraclass correlation [49,50].

## Results

Socio-demographic characteristics of the respondents are presented in Table 1. The participants ranged in age from 19 to 67 years, with a median age of 42 years. The majority was male (68%) and there were more heterosexual (59%) than homosexual, bisexual, or transgendered participants. Education levels were relatively low with only about 15 percent having achieved University level or more. Monthly income ranged from 0 to 4,790 USD (median 365 USD) with the majority classified in low socio-economic status based on the asset index. The sample was about evenly divided between those with or without a steady sexual partner. Of those with steady partners, only 13 percent were also HIV positive. About half the population did not have a child. Length of time on ARVs ranged from less than one year to 23 years, with a median of 6 years.

**Table 1:** Sample characteristics in study of PLWH on ARVs attending public clinics in Rio de Janeiro, Brazil (n = 632)

	**n**	**%**
**Gender**		
Male	429	68%
Female	196	31%
Transgender	7	1%
**Age [range: 19–67 years; median: 42]**		
19-35	153	24%
36-41	139	22%
42-47	172	27%
48-67	168	27%
**Race**		
White	169	27%
Non-White (Black, Brown, Other)	463	73%
**Education**		
Primary or less	278	44%
Secondary	262	41%
University or more	92	15%
**Sexual Orientation**		
Heterosexual	374	59%
Other (Homosexual, Bi, Trans)	258	41%
**Relationship Status**		
No Steady Partner	325	51%
With Steady Partner	307	49%
**HIV Positive Partner**		
No	547	87%
Yes	85	13%
**Number of Children**		
0	312	49%
1	113	18%
> = 2	207	33%
**Religion**		
Catholic	245	39%
Evangelical	165	26%
Other (Espirita, Afro-Brasilian, other)	141	22%
None	81	13%
**Length of time on ARVs [range: 0–23years; median: 6]**		
0-1yrs	147	23%
2-6yrs	199	32%
7-23 yrs	286	45 %

Eight-four percent of respondents self reported adherence to all of their ARV doses in the last four days. About 85 percent said they followed specific dosing instructions all or a majority of the time in the last four days. When asked the last time they missed taking any of their medications, 32 percent of respondents said they had never missed a dose, 35 percent more than a month prior, while 21 percent missed a dose in the past week. Only 17 percent of the sample missed a dose on the weekend. The most recent viral load measurements from the past year were obtained from respondents’ medical records. Self reported adherence was significantly correlated with viral load data (Pearson r=.22; p<.0001). Those with undetectable viral loads were twice as likely to self-report perfect adherence to ARVs in the last 4 days as those with detectable viral loads (UOR 2.07 CI [1.33-3.22]).

Bivariate associations between the independent variables and ARV adherence are presented in Table 2. Of the socio-demographic variables, older age, having two or more children, and a high education level were significantly associated with adherence. Thirty-five percent and 70 percent of respondents presented symptoms of moderate to severe anxiety and depression, respectively. Only anxiety was significantly associated with adherence but depression was still included in the multivariate model. High social support was significantly associated with adherence. In the bivariate analysis of the economic variables, having savings and high asset levels were significantly associated but individual, household or types of income were not significantly associated with adherence. Gender was significantly associated with adherence.

**Table 2:** Bivariate and multivariate analysis of individual, relational, and environmental-structural variables on the outcome: adherence to ARVs (n = 632)

	**% Adherent**	**UOR**	**(95% CI)**	**p-value**	**AOR**	**(95% CI)**	**p-value**
**Individual variables**							
**Sociodemographics**							
**Age [range = 19-67 years; median = 42]**							
19-35	78%	1.00			1.00		
36-41	81%	1.20	(.67 - 2.12)	0.543	1.40	(.59 - 3.33)	0.451
42-47	87%	1.88	(1.04 - 3.38)	0.037	1.86	(.88 - 3.95)	0.104
48-67	85%	1.57	(.89 - 2.79)	0.122	1.52	(.72 - 3.22)	0.268
**Race**							
White	86%	1.00			1.00		
Non-White (Black, Brown, Other)	82%	0.72	(.44 - 1.89)	0.200	0.82	(.43 - 1.59)	0.562
**Education**							
Primary or less	80%	1.00			1.00		
Secondary	86%	1.52	(.97 - 2.39)	0.068	1.19	(.92 - 1.53)	0.187
University or more	88%	1.90	(.95 - 3.80)	0.070	0.89	(.58 - 1.36)	0.580
**Sexual orientation**							
Heterosexual	81%	1.00			1.00		
Other (Homosexual, Bi, Trans)	86%	1.42	(.92 - 2.20)	0.116	1.08	(.58 - 1.99)	0.817
**Relationship status**							
No Steady Partner	84%	1.00			1.00		
With Steady Partner	82%	0.85	(.56 - 1.29)	0.455	0.73	(.46 - 1.14)	0.167
**Partner HIV status**							
HIV negative	84%	1.00			1.00		
HIV positive	79%	0.71	(.40 - 1.26)	0.244	0.71	(.43 - 1.17)	0.178
**Number of children**							
0	85%	1.00			1.00		
1	89%	1.40	(.73 - 2.69)	0.315	2.29	(1.33 - 3.94)	0.003
>=2	78%	0.65	(.42 - 1.03)	0.066	1.25	(.78 - 2.01)	0.363
**Religion**							
Catholic	83%	1.00			1.00		
Evangelical	82%	0.94	(.56 - 1.59)	0.824	1.12	(.60 - 2.09)	0.731
Other (Espirita, Afro-Brazilian, other)	84%	1.03	(.59 - 1.80)	0.914	0.91	(.30 - 2.72)	0.862
None	84%	1.05	(.53 - 2.08)	0.886	1.04	(.47 - 2.32)	0.921
**Length of time on ARVs**							
0-1 yrs	80%	1.00			1.00		
2-6 yrs	85%	1.50	(.86 - 2.64)	0.155	1.53	(.88 - 2.65)	0.128
7-23 yrs	84%	1.30	(.78 - 2.17)	0.307	1.11	(.73 - 1.67)	0.629
**Psychosocial variables**							
**Depression**							
No (<11)	83%	1.00			1.00		
Depressed (>11)	83%	0.98	(.62 - 1.54)	0.932	0.89	(.51 - 1.54)	0.678
**Anxiety**							
No (<11)	86%	1.00			1.00		
Anxiety (>11)	79%	0.61	(.40 - .93)	0.021	0.76	(.51 - 1.54)	0.177
**Relational variable**							
**Social support**							
Low	76%	1.00			1.00		
Medium	82%	1.46	(.91 - 2.33)	0.119	1.47	(.93 - 2.31)	0.096
High	91%	3.13	(1.67 -5.88)	0.000	2.85	(1.50 - 5.41)	0.001
**Environmental-structural variables**							
**Asset index**							
Low (1-7)	78%	1.00			1.00		
High (8-23)	91%	2.89	(1.77 -4.73)	0.000	2.47	(1.79 - 3.40)	0.000
**Savings**							
No	81%	1.00			1.00		
Yes	91%	2.40	(1.30 -4.42)	0.005	1.81	(.93 - 3.53)	0.079
**Gender**							
Male	86%	1.00			1.00		
Female	76%	0.53	(.35 - .81)	0.004	0.58	(0.38 - .88)	0.011
Transgender	100%	Predicts perfectly			Predicts perfectly		

*UOR* Unadjusted Odds Ratio, *CI* Confidence Interval, *AOR* Adjusted Odds Ratio.

In the multivariate analysis, as presented in Table 2, the model included socio-demographic variables, depression and anxiety, social support, gender, and economic factors. Of the sociodemographic variables, those who had one child (AOR 2.29 CI [1.33-3.94]) were positively associated with adherence but the same association did not apply to those who had two or more children compared to those who had no children. Those with high social support were significant associated with adherence than those with low social support (AOR 2.85 CI [1.50-5.41]). Of the economic factors, having a high asset index was independently associated with adherence than a low index (AOR 2.47 CI [1.79-3.40]). Gender was significantly associated with the outcome, with women less likely to be adherent to ARVs (AOR 0.58 CI [0.38-0.88]).

## Discussion

Self-reported adherence was high and comparable to other studies in Brazil [8,36,51]. We found that women were negatively associated with adherence to ARVs in this setting. While SES factors have not been consistently associated with adherence, we found a high asset index to be significantly associated with adherence. In addition social support was found to be significantly associated with the outcome, which is consistent with research among PLWH in other settings. Sociodemographic factors have generally not been consistently associated with adherence; we only found number of children to be significant which will be discussed in relation to gender. These factors and their relationships, especially pertaining to the history of the epidemic in Brazil and particularities of the research setting, will be discussed.

Women are especially vulnerable to HIV and negative HIV-related outcomes such as biological factors due to toxicity and social factors due to gender inequality [52-54]. A recent study examined factors associated with ARV discontinuation or modification and found women had a higher hazard for short-term toxicity [55]. In addition, women were found to have more severe side-effects than men, leading to higher rates of ARV discontinuation among women. Furthermore, there is a “feminization” of the epidemic with the male to female ratio decreasing from 5.9:1 in 1989 to 1.4:1 in 2006, highlighting the importance of increasing focus of research and interventions on women in Brazil [56]. Women might also experience challenges in accessing ARVs due to lack of knowledge, stigma and discrimination [57,58]. The history of activism in the Brazilian AIDS community started within the gay community and focused on MSM early on in the epidemic [59]. Women have not had the same amount of support from NGOs and most of the attention in Brazil to date among women has been on prevention efforts, which has also been the case globally [60,61].

We found having one child was associated with being adherent, versus not having any children, but the same did not apply for those having two or more children. A study in the US found that the number of children living at home is associated with nonadherence, and caretaking of children is often the responsibility of women [62]. Upon further analysis, adjusting for additional life stressors, the study still found the presence of children and not other stressors associated with nonadherence [62]. It appears that women place others’ needs, especially their children’s, ahead of their own [62,63]. While having at least one child in our study is associated with adherence, it might be a proxy for something else, such as stability in their life, but more than one child may create an extra burden. The role of caretaking and number of children warrants further research with regard to their role on adherence to ARVs among women in Brazil.

The pattern of the Brazilian HIV epidemic seems to initially affect higher socioeconomic levels and then disseminate into poorer populations [1,64]. This can be seen in the difference between the southeastern region where the epidemic began and is now among the middle and lower class versus the northeastern region where the epidemic is more recent and is concentrated in higher socioeconomic populations [1,65]. Even within our lower income population, respondents with higher levels of assets were significantly more likely to be adherent. This signifies the need to understand how wealth plays a role in adherence even in the context of free health services and free medication. Socioeconomic factors often serve as a proxy for other barriers to adherence such as transportation costs, lack of time, difficulty obtaining health information, and discrimination [66]. Poverty increases vulnerability to HIV infection and can also make it harder to cope with a complex chronic infection [67].

Social support is a multidimensional construct that has been defined and measured in various ways, focusing on sources of support and satisfaction with the support received [31]. Our measure was heavily focused on the provision of tangible material support such as help with obtaining medicines, performing chores, or going to the doctor. This is key to understanding the kinds of support important in addressing adherence. Future research should examine potential differences in the kinds of support women receive versus men since women are often the caretakers and carry a larger burden of household responsibilities, while men might have more economic burdens. Again, since the epidemic has reached women more recently, their needs have not received the same attention as other populations. They may not have the same extra-familial support networks to go to for support as MSM have within AIDS activist organizations within a setting such as Rio de Janeiro. In addition, fear of stigma may inhibit accessing support from their networks [57,68,69].

There was a linear trend between social support and assets, where those with more assets also had more social support. It is possible that participants with higher levels of assets also have family and friends with higher levels of assets; better enabling them to obtain the necessary support when needed. It is possible that those who suffer material deprivation also do not have the ability to access the necessary kinds of support from their social surroundings due to the lack of available resources. This can be potentially explained through the components of social capital which can be seen as horizontal or vertical, where linkages with people who are of similar status or background is called bonding social capital (horizontal) and people who are of a different background or identity is called bridging social capital (vertical) [70]. Further research in the realm of social capital, where resources available to an individual come from being a part of social networks, could help in understanding the relationship between assets and social support to develop care and support interventions among PLWH in lower income settings.

Anxiety and depression were not significant in the final model but the high prevalence among PLWH in this setting warrants further research. A recent study in Brazil using the same depression and anxiety measure but at a higher cutoff point to determine severe cases of depression and anxiety, found a high prevalence of anxiety (35.8%) and depressive (21.8%) symptoms among patients before initiating ARVS, which increased (51.5% and 40.6%, respectively) in the 175 day follow-up time period [39]. In addition, they found severe anxiety was significantly associated with non-adherence but not severe depression. We found similar anxiety levels in our study but a much greater prevalence of depressive symptoms (70%). This may be due to the longer average treatment duration among our study population since depressive symptoms increased in the aforementioned study over time.

### Limitations

Adherence is a dynamic behavior but our analysis is only cross-sectional; therefore we are only capturing one moment in time hence we cannot establish temporality in our associations. While self-report tends to overestimate adherence levels, it is reliable and tends to indicate average adherence [19]. The AGTC questionnaire has been tested in multiple settings and in Brazil as well. In addition, the self-report of adherence in this study was significantly correlated with the viral load data from their medical records. We attempted to decrease social desirability bias with a statement commenting on the difficulty of adherence before asking our questions. We also tried to limit recall bias by asking about adherence in the previous four days. The findings from this study come from regular clinics, where most Brazilians access care across the different regions, and not referral centers, where most of the studies in Brazil are conducted. Therefore these findings are more generalizable than other studies in Brazil but since Rio de Janeiro is the second largest city in Brazil, these findings should not be generalized to either poorer areas inside Brazil or low-income countries.

## Conclusions

This research highlights the importance of examining the multiple levels of influence on ARVs adherence. While the population had a high adherence rate, women were negatively associated with adherence compared to men, highlighting the gendered dimensions of providing effective care and support to PLWH. Even within a relatively poor population, having more wealth was associated with adherence. It is important to understand how wealth plays a role in adherence even in the context of free health services and medication, and in relation to social support. Intervention research in this and similar settings should address and evaluate the impact of attending to both gender and economic inequalities to improve ARV adherence and relational areas such as the provision of social support.

## Competing interests

The authors declared that they have no competing interest

## Authors’ contributions

FIB, MM, and DK designed the study. NB was the field coordinator and data manager. HH contributed to the questionnaire and field supervision. HH performed the data analysis and manuscript preparation. DK, PJW and PJS provided contributions into the interpretation of the data and results. All authors read and approved the final manuscript.

## Pre-publication history

The pre-publication history for this paper can be accessed here:

http://www.biomedcentral.com/1471-2458/13/574/prepub
